# FDIPP: False Data Injection Prevention Protocol for Smart Grid Distribution Systems †

**DOI:** 10.3390/s20030679

**Published:** 2020-01-26

**Authors:** Hosam Hittini, Atef Abdrabou, Liren Zhang

**Affiliations:** 1 Department of Electrical Engineering, UAE University, Al-Ain, Abu Dhabi 15551, UAE; 200834705@uaeu.ac.ae; 2 College of Physics and Electronic Science, Shandong Normal University, Jinan 250014, China; lirenzhang@sdnu.edu.cn

**Keywords:** smart grid, distribution system, security, integrity, communication architecture

## Abstract

In this paper, a false data injection prevention protocol (FDIPP) for smart grid distribution systems is proposed. The protocol is designed to work over a novel hierarchical communication network architecture that matches the distribution system hierarchy and its vast number of entities. The proposed protocol guarantees both system and data integrity via preventing packet injection, duplication, alteration, and rogue node access. Therefore, it prevents service disruption or damaging power network assets due to drawing the wrong conclusions about the current operating status of the power grid. Moreover, the impact of the FDIPP protocol on communication network performance is studied using intensive computer simulations. The simulation study shows that the proposed communication architecture is scalable and meets the packet delay requirements of inter-substation communication as mandated by IEC 61850-90-1 with a minimal packet loss while the security overhead of FDIPP is taken into account.

## 1. Introduction

Smart grids represent the next generation of power systems. They aim at turning the conventional power grid into a smart one using advanced information and communication technologies. Besides, smart grids integrate the usage of solar/wind energy in the power grid in the form of distributed energy generators changing the normal radial direction of the power flow. Thus, implementing smart grids is envisioned to increase the efficiency and reliability of legacy power grids. However, realizing the technical goals of smart grids requires efficient machine-to-machine (M2M) communication between intelligent devices, which implies an increased risk of communication and information security vulnerabilities. Exploiting such information system vulnerabilities is much more critical than exploiting vulnerabilities in conventional power networks. A successful attack on a smart grid can cause a power outage for large areas and, in turn, results in substantial financial losses.

The distribution system is an essential part of any power grid. It is responsible for delivering power to end consumers through two types of distribution substations; namely, primary substation (PS) and secondary substation or (SS). The latter connects consumers to a primary substation, whereas the former is connected to the transmission system. Every group of secondary substations is connected to one primary substation. Since secondary substations are responsible for distributing electricity to the consumer, each distribution system typically contains a huge number of secondary substations, which are highly distributed by nature. These substations are anticipated to use different kinds of sensing equipment in future distribution systems such as phasor measurement units and intelligent electronic devices. Therefore, they are anticipated to generate large M2M data volume, which poses a significant challenge to securing the underlying communication network architecture.

To increase the security of smart grids, the national institute of standards and technology (NIST) has developed cybersecurity development guidelines for communication interfaces between different smart grid domains [1]. The document shows a remarkable effort that can significantly reduce vulnerabilities. It has defined cyber-security requirement development guidelines for communication interfaces between different smart grid domains. The document is discussed in more detail in Section 2.1. Indeed, security functions should not hinder the ability of the network to transfer data timely and reliably. In the distribution system domain, the large number of nodes and their sparse nature, make wireless communication an attractive means to connect different entities of this domain. On the other hand, it makes enforcing security much harder, because intruders can gain physical access effortlessly, considering data is transferred over the air.

Acquiring the correct data from secondary substations with appropriate commands is vital for future smart grid operation. However, using M2M wireless communications in smart grids makes spoofing, alteration, replication, duplication a simple task. Receiving wrong information from secondary substations may result in making inappropriate decisions, and sending wrong operational commands, which may cause severe consequences. Researchers have identified many security attacks, which can cause fire, hurt people, or even cause blackouts for a whole city. For instance, authors of [2] describe an attack that results in preventing legitimate status messages from being delivered to the control center. In [3], the authors discuss a false data injection attack on the state estimation system, which is supposed to provide an accurate estimate of the power system status. Furthermore, using certain communication technologies such as WiFi or ZigBee in a smart grid makes it vulnerable to different security attacks that specifically address these technologies. Furthermore, key management among a vast number of nodes may lead to a negative impact on data transfer performance if the network architecture and key management technique are not designed with scalability in mind.

Thus, this work focuses on two main security aspects of smart grid distribution systems, namely, data confidentiality and integrity. Here, integrity includes both data integrity and system integrity. Altering the content of a transmitted packet is considered a violation of data integrity, whereas having a rogue node capable of injecting data is considered a violation of both data and system integrity. Moreover, authentication is taken into account as it improves system integrity by preventing unauthorized users from accessing the system assets.

The contributions of this paper are two-fold. First, the details of a NIST guidelines-compliant security-aware smart grid communication architecture for distribution systems are introduced (The proposed communication architecture is presented in part in the proceedings of IEEE IIT 2016 [4]). The security features of the proposed architecture are analyzed in terms of NIST security requirements [1]. Second, we propose a security protocol (FDIPP) that completely fits the underlying communication architecture and also adheres to NIST guidelines. The proposed protocol offers data and system integrity, in addition to confidentiality and authentication. Moreover, a key management procedure that accompanies the proposed protocol is also presented. Furthermore, the performance of the proposed communication architecture and protocol (including the key management procedure) in terms of end-to-end packet loss and delay is investigated using computer simulations against the communication requirements mandated by IEC 61850-90-1 [5].

The rest of this paper is organized as follows. Section 2 covers the literature review and related works. Section 3 presents the communication system architecture under study and discusses its alignment with NIST cyber-security guidelines. The full operation of the FDIPP protocol is described in Section 4 including the key management algorithm and security analysis. Section 5 addresses the performance evaluation of the proposed architecture and FDIPP. Finally, Section 6 concludes this work.

## 2. Literature Review

This section first covers a general background about NIST smart grid security guidelines and communication requirements for smart grid distribution systems. After that, the most relevant research works in the literature are introduced.

### 2.1. NIST Smart Grid Security Guidelines

The National Institute of Standards and Technology developed a conceptual architecture for smart grids. According to this model, the smart grid is made of 7 domains [6]. Here, we focus on the distribution domain, which interacts with other domains, such as Operations and Customer domains. The components of the distribution domain are as follows [1]:

- Distribution Sensors: Devices that measure physical quantities and send them as digital signals to be used by other actors in the system.
- Remote Terminal Units (RTUs) and Intelligent Electronic Devices (IEDs): Receive information from various sensors and send commands accordingly.
- Distribution Data Collector: A system that collects data from different sources and modifies or transfers these data.
- Distributed Intelligence Capabilities: Autonomous applications that operate separate from centralized control to increase responsiveness and reliability of the system.
- Geographic Information System: A management system that provides asset information and status for other advanced applications.
- Field Crew Tools: Maintenance hand-held tools that are used for field engineering.

**Figure 1:**
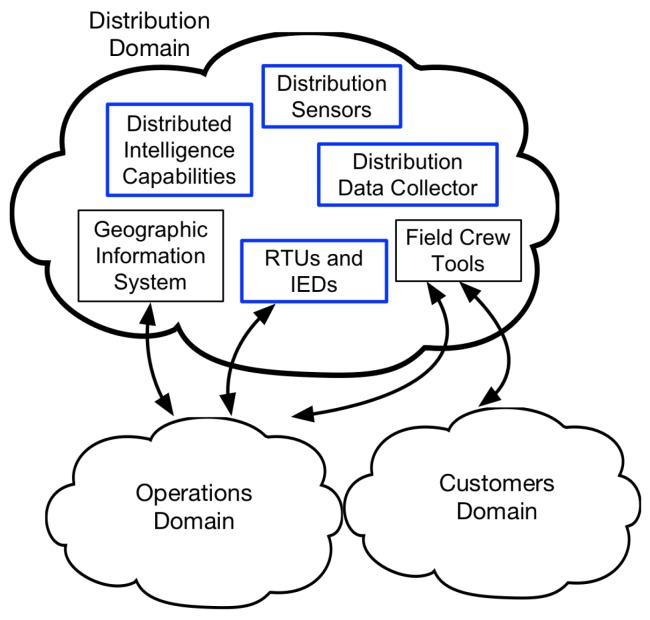
Distribution System Components.

The described actors are shown in Figure 1. Geographic Information System and Field Crew Tools are out of the scope of this work.

To secure different smart grid domains, NIST has developed a comprehensive set of guidelines for smart grid cyber-security in [1]. These guidelines represent the cyber-security requirements that guarantee (if met) securing smart grid operation and data transfer as in the following.

- *Concurrent Session Control*: This requirement mandates that the number of concurrent sessions should be limited.
- *Remote Session Lock and Termination*: This requirement highlights the need to define an inactivity period that results in a session timeout. After this period, users and devices need to re-authenticate to be able to activate the session again.
- *Permitted Actions without Identification or Authentication*: This implies defining access levels to users based on their roles in the system.
- *Remote Access*: Remote access should be disabled by default, and only enabled when required, approved, and for the required time only. All methods of remote access should be managed, authorized, and monitored.
- *Wireless Access Restrictions*: All wireless communication devices should be authenticated. Wireless data transfer should be encrypted. A wireless intrusion detection system (WIDS) should be in place.
- *User Identification and Authentication*: This requirement highlights that all system users should be uniquely identified and authenticated using multi-factor authentication.
- *Device Identification and Authentication*: An up-to-date list of authentic devices with their details should be prepared and securely stored. Authentication of all devices should be enforced using bi-directional authentication through an authentication server.
- *Denial-of-Service Protection*: This requirement aims to ensure that all nodes in a smart grid information system can mitigate the effect of DoS attacks.
- *Boundary Protection*: It addresses defining internal and external boundaries of the smart grid domain of interest and controlling communication functions at the defined boundaries. This includes allowing communication to external networks only through protected interfaces and limiting the number of these interfaces.
- *Communication Integrity*: The goal of this requirement is to warrant that a smart grid information system protects the integrity of data in all communication functions.
- *Software and Information Integrity*: It aims at detecting any unauthorized changes to information and software applications.

### 2.2. Distribution System Communication Requirements

Communication between substations in a distribution system has specific delay requirements based on the application type. These requirements are defined as in IEC 61850-90-1 as shown in Table 1 [5,7].

Messages of Type 1A have stringent latency requirements as they are used mainly for protection and fault isolation. Type 1B messages are used for traditional communications between different systems. Type 4 messages is used to transfer raw data, whereas Type 2 is mainly for monitoring applications. Finally, the usage of Type 3 and Type 6 messages is restricted to less critical messages such as low-speed data and file transfer. Admittedly, the latency requirements of different message types mandate designing a communication network with sufficient radio resources to meet the stringent ones especially. However, securing such a network adds an overhead, which consumes a part of the network bandwidth.

Therefore, the design of a security-aware smart grid distribution system communication architecture should: (i) have sufficient resources to meet the latency requirements of IEC 61850-90-1 with easy and low cost deployment, (ii) follow the NIST security guidelines, and (iii) guarantee a minimal operation overhead of its security protocols/mechanisms that does not violate the aforementioned latency requirements of different application types.

### 2.3. Related Works

The requirements and the design of a communication architecture for smart grids have drawn the attention of many researchers. The authors of [8] highlight the general characteristics and high-level requirements for smart grid communications, but no specific proposal for architecture is provided. In [9,10,11], possible communication technologies and communication requirements for smart grids are discussed without a focus on security-aware architectures. In [7,12], the authors survey cyber-security requirements and threats for smart grids. They also introduce an evaluation of these threats in different scenarios. A privacy-aware smart grid communication architecture is presented in [13]. The proposed architecture mainly targets the secrecy and anonymity of customers. Zhang et al. in [14] define information security risks and requirements for smart grids. They propose a high-level security framework to support smart grids, but it is not based on a specific architecture. The authors of [15] propose a general decentralized information infrastructure for smart grids over IP networks. The proposed infrastructure does not address a specific domain (e.g., the distribution system) or security protocols that cover specific security services over wireless networks.

In the literature, several research works investigate the detection of security attacks against data integrity. The authors of [16] target the data injection attacks on energy management systems (EMS) of power grids. They propose an algorithm that finds the minimum number of meters that will result in an unobservable attack if compromised, but they do not provide countermeasures. The work of [17] provides an algorithm to detect integrity attacks on smart metering infrastructure (SMI) if less than 5 meters are compromised and proposes a countermeasure based on state estimation. However, data injection can happen before the attack is detected, and the possibility of compromising more than 5 smart meters is not covered. In [18], a homomorphic signature for aggregated data is introduced. The proposed method is computationally inexpensive, but it does not cover system integrity. Guo et al. in [19] use historical data to find any inconsistency. A patient attacker can modify the data slowly enough to make it undetectable over a long time. The authors of [20] propose sending the data over a high-speed IP network while sending a watermark over a low bandwidth secure network. The number of nodes in a smart grid distribution system may limit the scalability of this solution. Yang et al. in [21] introduce a model-based detection scheme for data integrity attacks against smart metering infrastructure.

Other research works provide defense techniques against data modification due to integrity attacks. For instance, in [22], the authors present a parametric feedback linearization control scheme to stabilize the power system after a physical disturbance caused by a cyber-attack on data integrity. Yang et al. [23] propose a defensive strategy for integrity attacks against optimal power flow. The authors of [24] propose preventing pollution attacks against smart meter data collectors by a scheme that is based on proxy re-encryption and homomorphic authenticators. In [25], the authors propose a deep-Q-network scheme to learn the optimal strategy to defend the power grid against integrity attacks. The authors of [26] propose to protect smart grid state estimation from data manipulation using blockchain technology by detecting misbehaving nodes. Garg et al. in [27,28] introduce lightweight authentication schemes for resource-constrained smart meters and generally for Internet-of-things devices, respectively. Other authors, such as in [29], propose a privacy-preserving scheme for resource-constrained smart meters.

General security aspects (e.g., confidentiality, authentication, key management) of distribution system automation have been addressed in different parts of IEC 62351 standard [30]. Several research works focus on intrusion detection of distribution system automation, such as [31,32], whereas other works target anomaly detection [33]. Authentication of Modbus transactions and distributed network protocol 3 (DNP3) broadcast messages are addressed in [34,35], respectively.

**Figure 2:**
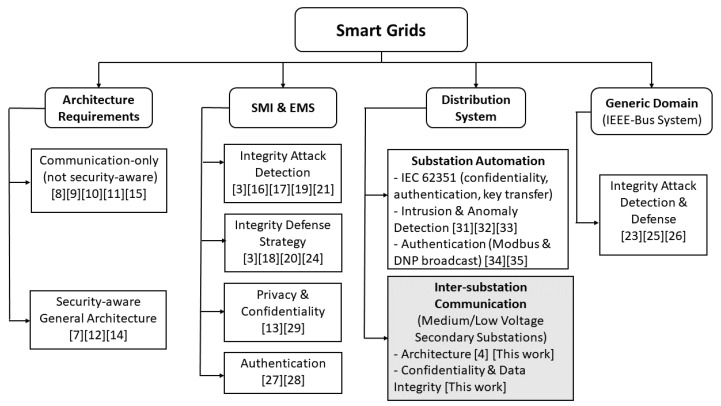
Related works categorized by research areas.

Figure 2 reveals the aforementioned related works classified according to their target research areas. The focus of this work is on the distribution system, particularly the inter-substation communication among medium/low voltage (e.g., 11/0.24 kV) secondary substations and between these substations and primary medium voltage substations (e.g., 33/11 kV). The number of secondary substations is typically vast as they are connected directly to consumer premises, while the delay constraint for reporting some data types is stringent as mandated by IEC 61850-90-1. Current SCADA systems normally do not monitor secondary substations (the low voltage network) [36,37]. Instead, they focus on monitoring the primary substations and the feeders connected to them. Smart grids are anticipated to accommodate distributed generators, which can be connected to secondary substations, and hence require data reporting to/from these substations. Our research work focuses on preserving privacy and integrity of inter-substation communication of the low voltage network, and hence it addresses challenges that are fundamentally different from the ones targeted by other research works focusing on other systems. For instance, research works targeting SMI or EMS (e.g., [16,17,21,24,27,28]) do not address strict delay requirements (in the order of tens of milliseconds) since smart meters mainly report usage data. The proposed research also has a different focus from IEC 62351 standard or the research works that address substation automation (e.g., [31,32,33,34,35]) since they tackle the security of the communication network and the devices used by primary distribution substations for automation purposes but not the inter-communication between secondary and primary distribution substations. Furthermore, some of the aforementioned research works target integrity attacks and defense techniques for smart grids, but the proposed schemes, such as [23,25,26], are applied on IEEE test bus systems (e.g., IEEE 30-bus systems) which neither capture the scale of the smart grid distribution system, in terms of the number of secondary substations, nor the impact of security-related functions on the performance of the underlying communication network.

To the best of our knowledge, no other research work in the literature addresses a security-aware communication architecture specifically designed for the distribution system domain of smart grids following the NIST guidelines and focusing on the requirements of inter-substation communication of the power distribution network. In addition, this work offers a confidentiality-and-integrity-preserving security protocol that is adaptable to the nature of the distribution system and has a minimal impact on the performance of the proposed architecture in terms of packet transfer delay and loss.

## 3. Communication Architecture Based on NIST Security Guidelines

This section presents the details of a communication architecture for smart grid distribution systems that is aligned with NIST security guidelines. Furthermore, the section highlights the role of the proposed protocol (FDIPP) in making this architecture meet some of NIST critical security requirements.

### 3.1. Network Configuration

Transferring a conventional power grid to a smart grid necessitates achieving a reliable and secure data transfer among many entities of the distribution system of the power grid. The ultimate goal is to achieve a fully automated operation of the system that includes many critical functions such as fault isolation, active monitoring, decentralized control, power network restoration, and reconfiguration. Indeed, this requires a communication architecture that is compatible with the current radial power flow and also supports the bi-directional power flow, which is anticipated to be widely spread in the future smart grids due to the existence of distributed generators (solar cells and wind turbines).

Consequently, any proposed communication architecture should be flexible and scalable to adapt to any change in the distribution system either from the low-voltage side (the consumer side), such as installing a photovoltaic panel in consumer premises or commissioning phasor measurement units (PMUs) inside the distribution network.

The communication architecture is hierarchical, where the control center lies at the top of the hierarchy. The network is divided into clouds, where each cloud is responsible for some functionality as in the following.

**Figure 3:**
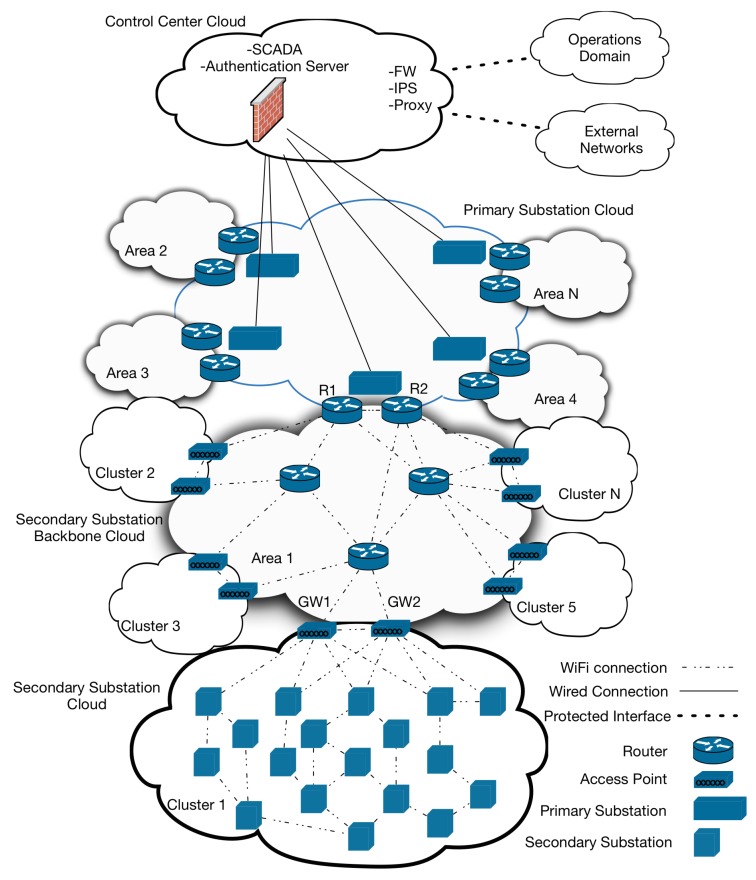
Security Aware Distribution System Architecture.

- **Control Center Cloud (CCC)**: It contains the supervisory control and data acquisition (SCADA) servers that are used to monitor and control substations and the authentication server. Besides, the CCC has managed network interfaces to allow communication to other domains or external networks. Thus, data communication through these interfaces is performed via a proxy, protected by a firewall (FW), and an intrusion prevention system (IPS). The communication inside the CCC usually uses a high-speed wired communication network.
- **Primary Substation Cloud (PSC)**: This cloud contains all the primary substations in the distribution system either high/medium voltage substations such as the 132/33 kV or medium voltage substations 33/11 kV in the European systems. These substations belong to different geographical areas. For instance, the high/medium voltage substations are typically close to the transmission system, whereas the medium voltage substations are directly connected to the medium/low substations (11 kV/0.24 kV) to feed the consumer premises. High and medium voltage primary substations (i.e., 132/33 kV and 33/11 kV, respectively) can communicate with the CCC and each other through their cloud by means of wired communication given their number and the distance between their sites. In Figure 3, only the links to the CCC are shown. The current SCADA systems do not monitor the medium/low voltage substations (secondary substations), but this is essential in future smart grid distribution systems, especially with the existence of distributed generators, which are typically connected from the low voltage side. Thus, for the sake of achieving active distribution network management, each medium voltage substation (33/11 kV) should be able to monitor and provide control features to the secondary substations connected to it. Therefore, in the proposed architecture, each primary substation is connected to several secondary substation clusters or clouds (SSCs) based on their geographic locations. The connection is made through a secondary substation backbone cloud, which is a wireless mesh backbone. The number of secondary substations in each SSC can be determined based on the requirements of smart grid control applications that the distribution network operator plans to run and the needed security level as discussed in Section 5.
- **Secondary Substation Backbone Cloud (SSBC)**: This cloud is a mesh network that connects each primary substation wirelessly to its SSCs via two routers (e.g., R1 and R2 in Figure 3 for redundancy). Each SSC is connected to the SSBC using also two gateways (e.g., GW1 and GW2 in Figure 3). The two gateways are the routers of the closest secondary substations to the SSBC backbone routers, but each one has an extra responsibility of forwarding the traffic of the SSC cloud to the rest of the network. The SSBC routers form a WiFi mesh network that relies on the IEEE 802.11s protocol [38]. However, end-to-end data transfer authentication, privacy, and integrity are achieved through the FDIPP protocol as described in Section 4. Thus, the SSBC can work with any on-demand ad-hoc or mesh routing protocol that does not have information security features.
- **Secondary Substation Cloud (SSC)**: It connects secondary substations to one another, to their respective primary substation (via the SSBC), and ultimately to the CCC to allow exchanging sensed data and/or commands. Each SS in an SSC is equipped with a WiFi router that has one wireless network interface. Secondary substations can communicate with one another and to the SSC gateway using any on-demand ad-hoc or mesh routing protocol as FDIPP offers privacy, authentication, and integrity for data transfer between them. Moreover, the secondary substation routers run host-based firewalls to protect the smart substation devices, which send their data through these routers.

### 3.2. Cyber-Security Awareness

In this section, the security awareness features for the proposed architecture are summarized. They are based on smart grid security guidelines document [6] developed by NIST. Each one of the following subsections represents one of the NIST guidelines.

#### 3.2.1. Concurrent Session Control

In the proposed architecture, this requirement is enforced at the CCC to be equal to the number of primary substations since each one can establish one session at a time to report status to the CCC. The limit on the concurrent number of sessions for a primary substation can be set as the number of the secondary substations under its control assuming in an emergency or an outage scenario all secondary substations in a cloud may be reporting status to the primary substation connected to the cloud. Consequently, the maximum number of concurrent sessions a gateway can forward is equal to the number of secondary substations in the SS cloud that are connected to this gateway. This directly leads to controlling the number of concurrent sessions that can be forwarded over the SSBC as it is related to the number of connected gateways.

Secondary substations can set the number of concurrent sessions to the number of first-hop and second-hop neighbors or based on how the grid is connected. Since a secondary substation can open a data session or a forwarding session, the number is expected to be variable based on how far the secondary substation location is from the primary substation.

#### 3.2.2. Remote Session Lock and Termination

Remote session termination can be met in a distributed fashion in the proposed architecture by defining an inactivity timer that leads to terminating inactive sessions that are initiated from secondary substations and primary substations. The same can be applied to gateways and SSBC routers if no packets are received from a particular session for a long time. Furthermore, an automatic session lock mechanism shall be enforced in all computing nodes that can be accessed by a human at the CCC. This also applies to SSBC routers, gateways, primary, and secondary substation devices.

#### 3.2.3. Permitted Actions without Identification or Authentication

The CCC contains an authentication server that also takes care of access control levels. This allows giving different access privileges (permitted actions) to different system users (e.g., SCADA operators and system admins) based on the type and location of the node they are trying to access (e.g., IED, RTU, a router, or a gateway).

#### 3.2.4. Remote Access

In the proposed architecture, remote access is allowed from the CCC to all primary and secondary substations in addition to gateways and backbone routers. This happens immediately after the successful FDIPP protocol authentication and the secure connection establishment between the CCC and these nodes. After FDIPP authentication phases are performed, remote access becomes also allowed from a primary substation to all the secondary substations under its control to provide sufficient redundancy in case the communication is not possible with the CCC. No remote access is allowed from any other point in the network.

#### 3.2.5. Wireless Access Restrictions

Since the proposed architecture mainly depends on wireless communications, all wireless network equipment (SSBC routers, gateways, secondary substations) is authenticated using the FDIPP protocol. Moreover, FDIPP protocol guarantees the secrecy and integrity of data transfer using encryption and hashing as discussed in Section 4. WIDS may not be used due to its implementation cost given the large scale of the network in terms of the number of nodes and coverage area. Instead, the FDIPP protocol ensures mutual peer authentication between communicating secondary substations and SSBC routers, whenever packet data transfer or forwarding occurs to eliminate possible attacks using rogue routers or APs. Furthermore, FDIPP peer authentication phase depends on a node authentication phase, which authenticates every single node in the network with the CCC authentication server. The hierarchical nature of the network allows the CCC and primary substations to quickly locate any detected wireless communication disruption due to a DOS attack.

#### 3.2.6. User Identification and Authentication

The centralized authentication server at the CCC allows all users to use multifactor authentication to access any device in the network based on the permitted access level. Different methods are available such as using a combination of a radio frequency identification (RFID) card and a device access password. This can be realized, for instance, by protecting system devices such as WiFi routers, IEDs, RTUs in tamper-proof enclosures, which can be opened once an RFID reader authenticates the user.

#### 3.2.7. Device Identification and Authentication

The requirement can be met by (i) preparing and documenting a list of authentic assets with their details and (ii) enforcing authentication for all wireless devices using bi-directional node authentication through the authentication server at the CCC. The proposed communication architecture makes it easy to meet the first requirement as it is partitioned into clouds, which simplifies the identification of nodes, their types, roles, and location by using a systematic naming convention. The second requirement is realized by the FDIPP protocol, which authenticates each node with the CCC authentication server.

#### 3.2.8. Denial-of-Service Protection

The following features of the proposed architecture help in meeting Denial-of-Service protection requirement:

- If the communication with a gateway failed due to DoS attack, its redundant node can take over and deliver traffic.
- A DoS attack on an SSBC router can be automatically mitigated by selecting another path using the operating ad-hoc on-demand routing protocol.
- The routing protocol running on secondary substation routers can select another neighbor station for packet forwarding in case the communication with the currently selected neighbor is disrupted.
- ARP requests and responses can be replaced by populating the ARP cache of different nodes manually with their neighbors’ MAC addresses and corresponding IP addresses.
- Due to the periodic reporting nature of regular data traffic from primary and secondary substations, any communication disruption can be easily noticed at the CCC and primary substations, respectively.
- Applying defense-in-depth techniques in the CCC can protect from most attacks coming to or through the CCC, including DoS and distributed DoS (DDoS).
- All routers should be equipped with host-based firewalls.

#### 3.2.9. Boundary Protection

This requirement applies to the interface between the CCC and Operations System, and the interface between the CCC and external networks, as shown in Figure 3. Communication to other domains is done through protected managed interfaces only. The number of managed interfaces is limited to one interface per smart grid domain and one more interface to external networks.

#### 3.2.10. Communication Integrity

This requirement is met by using the FDIPP protocol, which provides authentication, encryption, and hashing to all data messages sent across the proposed network architecture as presented in Section 4.

#### 3.2.11. Software and Information Integrity

All devices such as sensors, IEDs, RTUs, and routers should be kept in tamper-proof containers that can be securely opened by authorized personnel by providing valid access cards (e.g., RFID cards) and a password to be able to access/configure the software running on these devices. Different access control levels should be assigned to retrieve sensitive information such as stored key material. Performing regular tampering checks every defined period should be done to ensure physical security. In addition, all nodes should utilize the principals of least privilege and role-based access control to minimize the chance of unauthorized changes to any software.

## 4. FDIPP Description

FDIPP is designed to provide confidentiality and integrity. It covers both system and data integrity as it protects against packet injection, duplication, alteration, and node replication. The protocol operation is divided into three phases, namely, node authentication, peer authentication, and data transfer. The first phase authenticates the wireless access of the node with the Authentication Server (AS), whereas the second authenticates peers, in the same network (cloud), with each other on-demand. For instance, if Node 1 intends to use Node 2 as its next hop, it initiates the mutual peer authentication phase with Node 2 after the wireless access authentication of both nodes is completed in the first phase. In the data transfer phase, the confidentiality and integrity of the system data are protected using symmetric encryption and hash-based message authentication code (HMAC).

The section first outlines the assumptions then provides the details of the operational procedure of FDIPP. This includes node wireless access authentication, peer authentication, and post-authentication data transfer phases. After that, formal FDIPP security analysis and the key management mechanism are also presented.

### 4.1. Assumptions and Notations

The operation of the FDIPP protocol relies on the following assumptions:

- All network cables that connect the primary substations to the Control Center are secure.
- The CCC is highly secure. It reduces the probability of compromise by employing defense-in-depth techniques such as next generation antivirus software (i.e., end point detection and response), intrusion detection systems, intrusion prevention systems, firewalls, multi-factor authentication, and access control.
- The communication link between a primary substation and the authentication server is physically secure. Furthermore, connecting a new primary substation to the authentication server is done via a secure process.
- All computers in any substation (either primary or secondary) are assumed tamper-proof. Likewise, all power station premises are assumed physically secure.
- There are private/public key pairs generated and stored at all devices at commissioning time.
- The AS keeps track of the authenticated nodes, their IP addresses, and the cloud they belong to. This helps in revoking access at any time if intrusion attempts are detected.
- The AS stores a database of a long term key $k_{i}$, a one time key $y_{i}$, and automatically generated password $p_{i}$ for every node *i* in the network. Furthermore, $k_{i}$, $y_{i}$, and $p_{i}$ are securely stored in each node *i* and become available at the commissioning time.
- Time is synchronized between all nodes.

The symbol $E_{K}\left(.\right)$ denotes encryption with the key *K*, H(.) denotes a secure hash function, and || symbol is used to represent concatenation throughout this work. The FDIPP operation described in the following sections assumes the usage of a hash function such as SHA or SHA-3 [39] with an output digest of 256 or 512 bits, a symmetric encryption such as AES-GCM (Galois/Counter mode) with a 256-bit key, and asymmetric encryption such as RSA-OAEP (optimal asymmetric encryption padding) with a 2048-bit key. Although the authors of [40] present multiple attacks on RSA, all of these attacks can be mitigated by ensuring physical security as in the assumptions mentioned above. Furthermore, the design of the FDIPP protocol allows the use of any cryptographic algorithm for symmetric encryption, asymmetric encryption, and hashing.

### 4.2. Node Authentication

**Figure 4:**
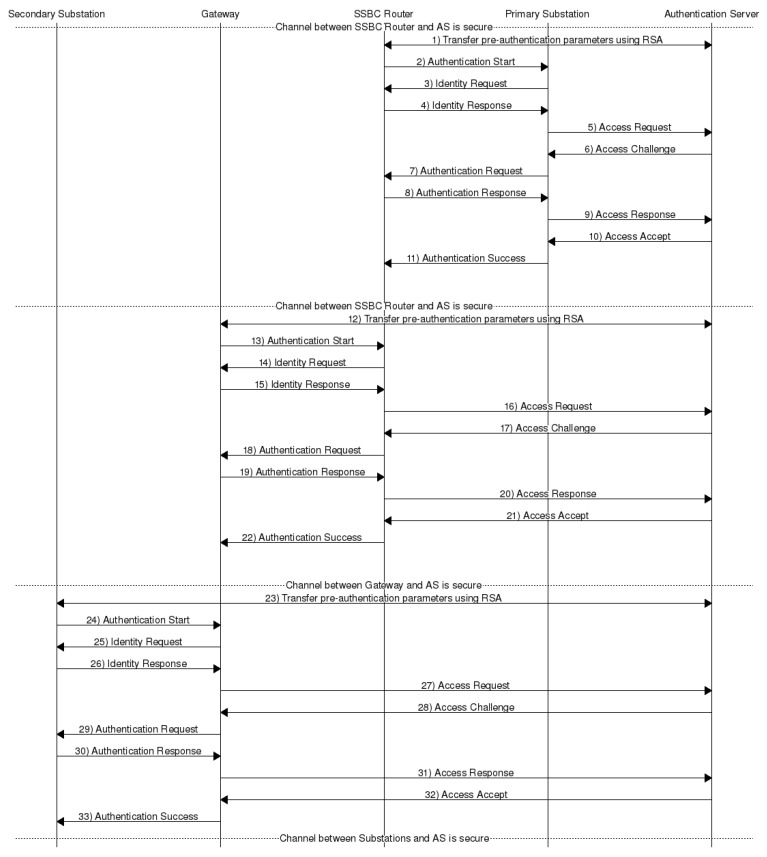
Node Authentication Sequence Diagram.

The node authentication phase is performed by three consecutive procedures across the whole network, namely, SSBC Router authentication, gateway authentication, and Secondary Substation (SS) authentication. Figure 4 shows the complete sequence diagram for node authentication in FDIPP. The three procedures are identical. Therefore, we only describe the steps of SSBC Router authentication phase below.

The EAP authentication mechanism proposed in [41] is integrated into this phase as it is designed to provide forward secrecy with low computation and communication cost. Moreover, this mechanism meets all the security requirements of RFC 4017 [41]. Thus, it is aligned to both the communication architecture requirements and the NIST guidelines mentioned in Section 2.1 and Section 2.2, respectively.

The following steps are performed first between the SSBC routers that are one hop away from the primary substation (PS) connected to their cloud. The objective is to establish a secure link between these SSBC routers (the supplicants) and the AS. Here, the primary substation plays the authenticator role since the channel between it and the AS is considered secure.

- Step 1: SSBC Router → PS: Authentication start.
- Step 2: SSBC Router ← PS: Identity request.
- Step 3: SSBC Router → PS: The router provides a temporal ID as the identity response. The sent message is $[E_{k⊗y}\left(RID\right),E_{k⊗y}\left(RID|\right|N_{R})]$ where $N_{R}$ is the router’s nonce.
- Step 4: PS → AS: The primary substation forwards $[E_{k⊗y}\left(RID\right),E_{k⊗y}\left(RID|\right|N_{R})]$ to the AS.
- Step 5: PS ← AS: The AS sends a challenge made of $H\left(N_{R}\right)⊗E_{k⊗y}\left(ASID|\right|N_{AS}||y{}_{N})$, where $N_{AS}$ is a nonce generated by the AS, $y_{N}$ is a randomly generated key, and $H(N_{R})$ is the hash of the router nonce.
- Step 6: SSBC Router ← PS: The PS forwards $H\left(N_{R}\right)⊗E_{k⊗y}\left(ASID|\right|N_{AS}||y{}_{N})$ to the router. The router will be able to extract $N_{AS}$ and $y_{N}$ by XORing the received message with $H(N_{R})$ again. Then decrypting $\left(ASID||N_{AS}||y{}_{N}\right)$. After that, it sets $y\leftarrow y{}_{N}$.
- Step 7: SSBC Router → PS: The router responds with $H(RID||p||y_{N})$.
- Step 8: PS → AS: The primary substation forwards $H(RID||p||y_{N})$ to the AS.
- Step 9: PS ← AS: If the hash was correct, the server sets $y\leftarrow y{}_{N}$ and sends Access Accept message. It also generates a unique session key $SK_{i-AS}$ for node *i*, a unique cloud key $CK_{Cj}$ (the key for the SSBC *j*), and forwards each one of them to the primary substation in a message as mentioned in Section 4.4.
- Step 10: SSBC Router ← PS: The PS sends Authentication Success message and forwards the messages containing $SK_{i-AS}$ and $CK_{Cj}$. Each key is 512 bits in length and divided in two equal parts (i.e., $SK_{i-AS}=SK1_{i-AS}\left|\right|SK2_{i-AS}$ and $CK_{Cj}=CK1_{Cj}\left|\right|CK2_{Cj}$); one is used for AES encryption, whereas the other half is used to generate an HMAC. After the successful authentication of an SSBC router, the channel between this router and the AS is considered secure. This allows the router to act as the authenticator for the SSBC routers that are one hop away from it (two hops away from the primary substation). The process then continues until the AS authenticates all the SSBC routers in the SSBC cloud.

Consequently, a secondary substation cloud gateway is authenticated the same way by the AS using an SSBC router that is one hop away from this gateway. Once the gateway is authenticated, a secure channel exists between the gateway and the AS. This allows the gateway to act as an authenticator to the routers located one hop away from the gateway in the SSC (secondary substation cloud) connected to it. The rest of the routers in the secondary substation cloud are authenticated in a hop-by-hop fashion similar to the process followed to authenticate the SSBC routers.

### 4.3. Peer Authentication

**Figure 5:**
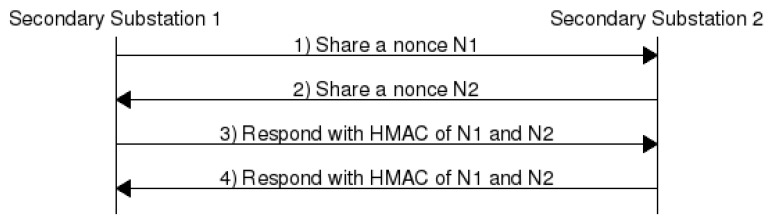
Peer Authentication Sequence Diagram.

Secondary substations can communicate with one another in the same cluster using single-hop or multihop communication based on the distance and transmission range. We assume here the usage of a widely adopted on-demand routing protocol such as ad-hoc on-demand distance vector (AODV) protocol for the communication among secondary substations. Although the authentication server authenticates all the nodes in a cloud, the nodes should authenticate each other before SS1 sends data to SS2 or uses SS2 as its next-hop packet forwarder. An example of the peer authentication method for secondary substations in a cloud *j* is shown in Figure 5 and the authentication method is described in details as in the following.

- SS1 → SS2: Secondary Substation 1 (SS1) sends its ID (SSID) and the nonce $N_{SS1}$ to Secondary Substation 2 (SS2) as $E_{CK1_{Cj}}(N_{SS1}||SS1ID)||H(E_{CK1_{Cj}}(N_{SS1}||SS1ID)||CK2_{Cj})$. SS2 stores the nonce $N_{SS1}$.
- SS1 ← SS2: SS2 responds with its ID and the nonce $N_{SS2}$ to SS1 as $E_{CK1_{Cj}}(N_{SS2}||SS2ID)||H(E_{CK1_{Cj}}(N_{SS2}||SS2ID)||CK2_{Cj})$. SS1 stores the nonce $N_{SS2}$.
- SS1 → SS2: SS1 responds with HMAC of both nonces in $E_{CK1_{Cj}}(H(N_{SS1}\left|\right|N_{SS2}||CK2_{Cj}))$. If the hash is correct, SS2 considers SS1 to be authentic.
- SS1 ← SS2: SS2 responds with HMAC of both nonces in $E_{CK1_{Cj}}(H(N_{SS1}\left|\right|N_{SS2}||CK2_{Cj}))$. If the hash is correct, SS1 considers SS2 to be authentic.

### 4.4. Key Management

Since the proposed architecture has a large number of nodes, key management is vital. Key management defines how keys are generated, distributed, and when they should expire. Three different types of keys are utilized in FDIPP. Asymmetric keys are used for exchanging identities and transferring symmetric key material. Symmetric keys are used for: (i) exchanging cloud-related control messages such as routing messages. These messages are encrypted and key-hashed, in each cloud, using a cloud key, and (ii) exchanging data/messages between two endpoints (two secondary substations, a primary and a secondary substation, the AS and a secondary substation, or an SSBC router and a gateway). This data is transferred encrypted and key-hashed using a session key.

Key management in FDIPP includes the following:

*The PKI System:*

1. Twelve public RSA keys of the AS are securely stored in all nodes. Each key has a minimum validity period of one month.
2. The AS also has twelve public keys for each node in the system. Each key should be used for a minimum period of one month.
3. Asymmetric RSA keys are changed every configured period in a predefined order.
4. RSA keys have to be physically replaced by authorized personnel every a minimum time of one year at all nodes in the system.

*Session and Cloud Keys:*

1. The session key $SK_{i-AS}$ is used to transfer messages between node *i* and the AS or the Control Center, where i∈{SSBC,Gateway,SSCloud}. Furthermore, the cloud key $CK_{Cj}$, the key for the cloud *j*, is transferred from the AS to node *i* in another message (the cloud here refers to either SSBC or SSC). The two keys are automatically sent from the AS to node *i*, after the Node Authentication phase is successfully done, by the message $E_{Pubi}(M_{AS}||H(M_{AS}\left|\right|k_{i}))$, where Pubi is the public key of node *i*, $M_{AS}=msg||N_{AS}\left|\right|SK_{i-AS}$ or $M_{AS}=msg||N_{AS}\left|\right|CK_{Cj}$ (based on the type of the key), and msg is the message body including message information such as the message type, node ID, and validity period. The keys have randomized validity durations and will be periodically resent by the AS prior to the end of their validity periods.
2. The session key $SK_{SSj-S}$ is used to transfer data messages between a secondary substation *j* (SSj) and a primary or another secondary substation (referred to as *S*). This key is to be created and transferred as follows:
  -$SS_{j}\to$ AS: The node sends an encrypted Key Request message $E_{SK1_{j-AS}}\left(M_{ss_{j}}\right)||H(E_{SK1_{j-AS}}\left(M_{ss_{j}}\right)||SK2_{j-AS})$, where $M_{ss_{j}}=msg\left|\right|N_{ss_{j}}$ and $N_{ss_{j}}$ is the node nonce.
  -$SS_{j}$← AS: The AS sends an encrypted Key Response message to $SS_{j}$$E_{SK1_{j-AS}}\left(M_{AS}\right)||H(E_{SK1_{j-AS}}\left(M_{AS}\right)||SK2_{j-AS})$, where $M_{AS}=msg||SK_{SSj-S}\left|\right|N_{AS}$.
  -*S*← AS: The AS sends the same message to the PS or the other SS (referred to as *S*) $E_{SK1_{S-AS}}\left(M_{AS}\right)||H(E_{SK1_{S-AS}}\left(M_{AS}\right)||SK2_{S-AS})$, where $SK2_{S-AS}$ is the session key between the primary or the other secondary substation and the AS.

This session key is supposed to have a short validity period. Renewal is following the same previously mentioned procedure. The AS monitors the frequency of Key Request message between an SS and the PS or between two secondary substations. Frequent communication mandates a long validity period to allow fast data transfer.

### 4.5. Post Authentication Data Transfer

After completing both node and peer authentication phases, secondary substations can exchange packets with each other and the primary substation. These packets can contain either routing messages or data. Routing messages are encrypted and signed with HMAC using respective cloud keys. For instance, routing messages between SS in $SSC_{j}$ and a PS are encrypted using the $CK1_{Cj}$ and signed with HMAC using $CK2_{Cj}$ in the form of $E_{CK1_{Cj}}\left(Rmsg|\right|N_{SS1})||H(E_{CK1_{Cj}}\left(Rmsg|\right|N_{SS1})||CK2_{Cj})$, where Rmsg is a routing message. The encryption key changes to $CK1_{SSBC}$ (and the hash key accordingly) as the packet traverses the SSC gateway. Data messages are sent between two endpoints encrypted and signed with HMAC using respective session keys (i.e., $SK_{i-AS}$ between a node and AS or CC and $SK_{SSj-S}$ for the communication between two SSs or an SS and PS).

### 4.6. Security Analysis

Evaluating newly developed security protocols plays a vital role in determining their trustworthiness. The authors of [42] compare multiple security evaluation approaches, namely, model checking or theorem proving, symbolic or cryptographic, and bounded or unbounded. In model checking, algorithms are followed to verify security, whereas proofs should be constructed to verify security in theorem proving. However, theorem proving requires significant experience and effort. Symbolic evaluation does not analyze the mathematical cryptographic basis of the protocol under study. Dolev–Yao model [43] is usually used for symbolic protocol representation and analysis. On the other hand, probability and complex theories are required for cryptographic analysis. Bounded security protocol analysis limits the number of concurrent protocol sessions an attacker can have. Increasing the number of these sessions may allow an attacker to manipulate replay messages and introduce new vulnerabilities in the protocol [42].

#### 4.6.1. Node Authentication

A security analysis of the Node Authentication phase can be obtained using theoretical proof following the details mentioned in [41,44]. Through this analysis, it can be proved that the Node Authentication phase provides mutual authentication, forward secrecy, and secure key exchange provided that the employed symmetric encryption is secure against adaptively chosen-ciphertext attack, which is the case with AES-GCM [45].

#### 4.6.2. Peer Authentication

Scyther [46] is a formal security analysis tool. It employs automatic security verification of protocols. Moreover, it supports bound and unbound parallel sessions. Scyther tool [46] fits the proposed protocol for two reasons. Firstly, it uses symbolic analysis, which implies that the cryptography used in the protocol is not analyzed and assumed perfect [46]. This aligns well with FDIPP because cryptographic algorithms are beyond the scope of this work. Secondly, it supports both bounded and unbounded analysis. Thus, it allows setting the maximum number of parallel sessions and identifies attacks within that bound. In the sequel, the tool configuration, security claims, and results are presented.

**Figure 6:**
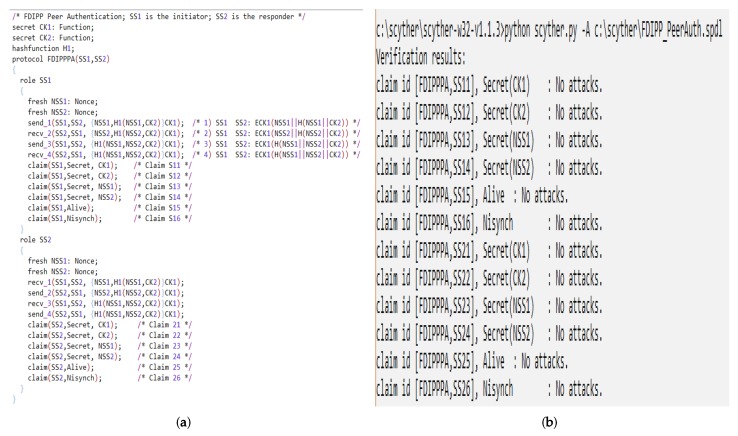
FDIPP Peer Authentication Analysis. (**a**) Peer Authentication analysis input. (**b**) Peer Authentication analysis result.

The tool is configured to assume CK1 and CK2 are secrets, whereas N1 and N2 are nonces that are incremented in every message. The Peer Authentication phase is provided as an input to the tool, as shown in Figure 6a. The tool analyzes the protocol against the following claims:

1. Secrecy: Secret information is not revealed to an intruder although the communication network is not trusted.
2. Aliveness: Communication partner is alive and able to initiate an event that the other partner can receive. For example, an intruder replaying messages sent earlier is considered a violation of the aliveness claim.
3. Synchronization: Communication parties are synchronized (i.e., if node A sends message 1 to node B, a response with message 2 is provided by node B). Synchronization covers both ordered and unmodified delivery of messages.
4. Agreement: Communication parties agree on the values of all variables transferred in the protocol.

The authors of [46] proved that achieving synchronization leads to achieve agreement also, but the opposite does not apply. The security analysis provided by the tool shows that the FDIPP Peer Authentication protocol is secure as all claims are verified as shown in Figure 6b.

## 5. Performance Evaluation

This section addresses the performance evaluation of the proposed architecture in terms of data transfer latency and packet loss while the FDIPP protocol is in operation. The simulation setup is introduced and the simulation results, which cover the SSC cloud and SSBC clouds, are discussed.

### 5.1. Simulation Setup

Since the primary substation cloud mainly depends on high-speed wired communications, the effect of FDIPP security features on packet transfer latency is assumed negligible. Thus, the simulation setup is divided into two parts, where each part represents a data transfer stage. The first stage covers the communication from a secondary substation (in an SSC) to the cloud gateway (another secondary substation in direct contact with a router in the SSBC). The second stage addresses the communication from the gateway to the primary substation that controls the cloud. The results of this stage (i.e., packet delay and loss) also apply to the CCC. Therefore, the total end-to-end packet delay is given as

(1) $$D_{e2e}=D_{s1}+D_{s2}$$

where $D_{s1}$ and $D_{s2}$ are the packet delay for the first and second stage, respectively.

(2) $$PLR_{e2e}=1-\left(1-PLR_{s1}\right)\left(1-PLR_{s2}\right)$$

where $PLR_{s1}$ and $PLR_{s2}$ are the packet loss ratio for the first and second stage, respectively.

The impact of the operation of the proposed protocol on the network performance in terms of packet loss and delay is comprehensively studied using the ns-2 simulator. In order to account for the effect of the FDIPP operation, the computer simulation of each stage is performed with and without key exchange. Moreover, different packet sizes are included to account for different data payloads and the overhead of security functions. However, authentication messages are not considered since they do not accompany data transfer. Thus, we assume that node and peer authentication phases are completed before the beginning of the simulation.

In the first data transfer stage, secondary substation routers in an SSC communicate over multi-hops with the cloud gateway using the medium access control (MAC) IEEE 802.11 protocol with a maximum transmission rate *R* of 54 Mb/s. In fact, this rate is similar to the expected achievable rate of relatively new standards, such as IEEE 802.11n, in outdoor scenarios, where the distance is large, and line-of-sight communication may not be possible to realize [47]. Each substation is assumed to send information at a rate of 10 messages per second. In the second stage, the SSBC routers form a mesh network backbone using IEEE 802.11n with an assumed maximum rate of 300 Mbps since the mesh backbone routers can be arbitrarily placed to be at a relatively close distance from one another with a line-of-sight transmission.

In both stages, the ns-2 simulation time is set to 60 s.The simulation parameters are mentioned in Table 2 including the IEEE 802.11 parameters [48]. At least 50 sample runs are considered for each variation of the parameters under study. In each sample, nodes are randomly placed over an area of 800 m × 800 m. Furthermore, data transfer start time is randomized among sending nodes.

### 5.2. Simulation Results for SS-Gateway Communications

**Figure 7:**
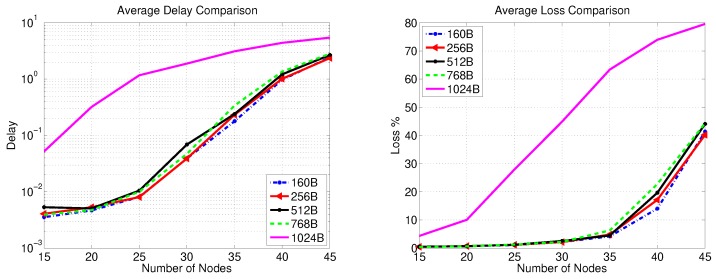
SS-Gateway communication performance (no key transfer).

**Figure 8:**
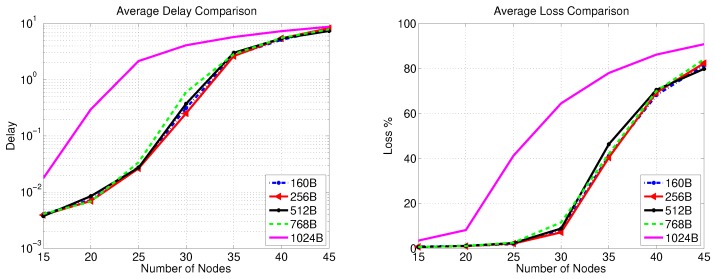
SS-Gateway communication performance (2048-bit key transfer).

The packet transfer delay and loss for the first data transfer stage are presented in Figure 7 and Figure 8 for no key exchange and 2048-bit key exchange, respectively. In each figure, the effect of varying the data volume is introduced by changing the data packet size from 160 Bytes to 1024 Bytes. Figure 7 shows the nominal performance when no key exchange is in place. The figure reveals that increasing the number of secondary substations in an SSC beyond 30 nodes leads to a packet loss that exceeds 2% irrespective of the data packet size except the case of 1024-Byte packets, where a significant packet loss increase happens with a lower number of nodes (15). Figure 7 also shows that a packet delay from 40–60 ms is observed for different packet sizes (except for 1024-Byte packets where it exceeds 1 s) when 30 secondary substations are simultaneously sending data with the aforementioned rate. The packet delay range decreases to below 10 ms for less than 25 secondary substations and the same packet sizes.

Figure 8 shows the network performance in the worst-case scenario of simultaneously communicating/updating the session key $SK_{i-AS}$ (encrypted using RSA-OAEP) between the AS and all secondary substations in an SSC at the same time. We assume here the usage of an HMAC with 256-bit digest, 512-bit encryption+HMAC session key, and 512 bits are dedicated to the nonce and the message body. The figure clearly shows the effect of the security overhead on packet delay, which varies from 300–500 ms for 30 SSs and from 20–30 ms for 25 nodes for all tested packet sizes except 1024 Bytes. Furthermore, the observed packet loss is around 1% or less for 20 nodes.

### 5.3. Simulation Results for Gateway-PS Communications

The data transfer in this stage is investigated for two scenarios. The first assumes that all the secondary substations belong to one SSC served by a single gateway. It is evident from the results of Section 5.2 that increasing the number of nodes (communicating with the gateway over a single radio channel) beyond 30 is not desirable. However, gateways can support two different channels. Thus, in the first scenario, we allow the input traffic of the gateway to come from a larger number of nodes. In the second scenario, we investigate the impact of using more than one gateway (or more than one SSC), where each gateway forwards the traffic of an SSC that has a smaller number of nodes than the first scenario. In both scenarios, the impact of transferring the largest security key on packet delay and loss is studied.

**Figure 9:**
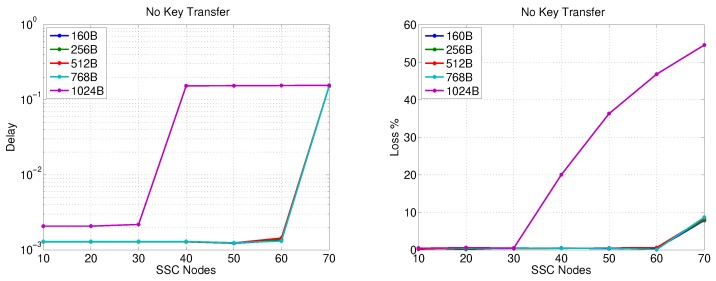
Gateway-PS communication performance for one SSC with different number of SSs (no key transfer).

**Figure 10:**
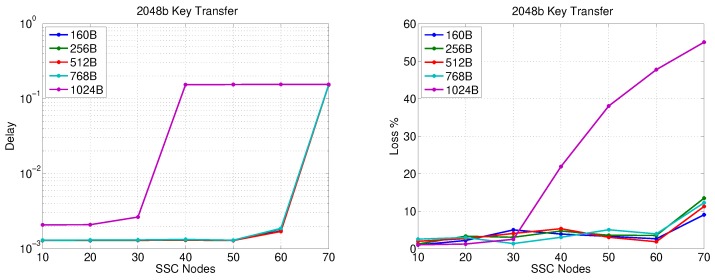
Gateway-PS communication performance for one SSC with different number of SSs (2048-bit key transfer).

Figure 9 depicts the packet delay and loss when there is a single gateway for an SSC cloud sending different traffic volumes (based on the number of secondary substations in the SSC) without key transfer. Apparently, sending the traffic of more than 60 secondary substations to a PS via one gateway leads to a rapid increase of packet delay and loss with increasing the number of SSs for all packet sizes except 1024 Bytes, where the packet delay and loss steeply increase after a traffic volume of 30 secondary substations. Figure 10 reveals that the transfer of 2048-bit key does not have a significant impact on packet delay compared with the results presented in Figure 9 for all packet sizes. However, the packet loss is increased to be around 4–5% as the gateway simultaneously handles the data and key material traffic for more than 20 secondary substations.

**Figure 11:**
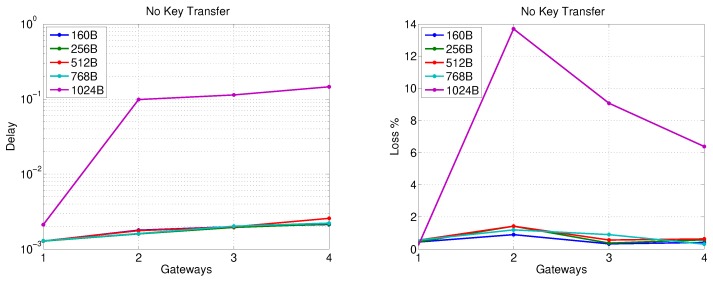
Gateway-PS communication performance for different number of gateways (no key transfer).

**Figure 12:**
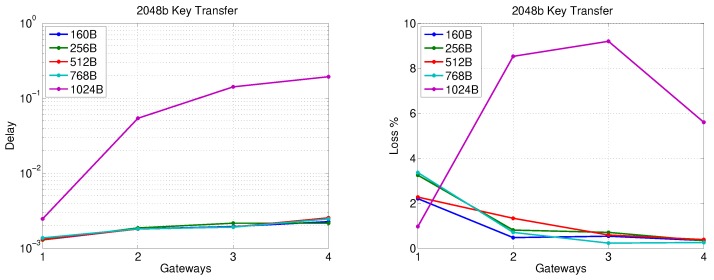
Gateway-PS communication performance for different number of gateways (2048-bit key transfer).

Figure 11 shows the packet delay and loss without key transfer as the number of gateways is varied from one to four where the total traffic load generated by the gateways under consideration is fixed and equivalent to the traffic received from 40 SSC nodes (e.g., for two gateways, each gateway serves 20 SSC nodes). The figure reveals that the delay for this packet transfer stage does not exceed 3 ms, while the packet loss is around 1% for different packet sizes (except 1024 Bytes). Figure 12 presents a comparison with Figure 11 in terms of packet delay and loss when the largest key size used by the FDIPP protocol (2048 bits) is being transferred. As depicted in Figure 12, key transfer has an insignificant effect on packet delay. However, the effect of the key transfer on packet loss is significant when one gateway is used for key transfer since it represents a bottleneck in sending data and receiving key material simultaneously. It is apparent from Figure 12 that packet loss decreases with increasing the number of gateways (i.e., smaller SSC clouds are used), while the same amount of traffic is pushed over the SSBC network.

### 5.4. Overall Performance Discussion

In this section, the FDIPP performance is discussed in terms of (i) packet transfer delay, loss, and the associated computational latency of security algorithms, and (ii) convergence or execution time for the Node Authentication and Peer Authentication phases.

#### 5.4.1. Packet Transfer Delay, Loss, and Computational Latency

The extensive simulation results of Section 5.2 and Section 5.3 reveal that during the transfer of a 2048-bit key, the traffic of 40 nodes in an SSC suffers from a substantial packet loss and delay at the gateway, if transferred to a single-transceiver gateway, due to the formation of a contention region around it. Furthermore, this traffic volume causes a packet loss of around 4% if transferred to the PS through the SSBC using one gateway.

Moreover, the results indicate that, during a 2048-bit key transfer, a traffic of 40 secondary substations can be communicated over the SSBC using two gateways, where each gateway supports one SSC, for a packet loss probability of around 1.5% for the SS-Gateway connection and almost the same probability for the Gateway-PS connection. On the other hand, the packet delay for the SS-Gateway connection is around 9 ms, whereas it is around 1 ms for the Gateway-PS connection. Using (2) and (1), this leads to an overall end-to-end packet loss probability $PLR_{e2e}$ of 3% and end-to-end packet delay of 10 ms, respectively.

Furthermore, the results reveal that transferring the traffic of 40 secondary substations during 2048-bit key transfer using four gateways, where each gateway supports a 10-substation SSC, leads to a packet loss lower than 0.5% for each of the SS-Gateway and Gateway-PS connections. This leads to an end-to-end packet loss probability of lower than 1% using (2). Moreover, the end-to-end delay does not exceed 7 ms from an SS to the PS for all packet sizes other than 1024 Bytes, which meets the most stringent delay constraint (e.g., Type 1A) as mentioned in Table 1.

It is worth noting that the above-mentioned packet delays are not significantly affected by the computational complexity of the employed cryptographic algorithms for two reasons. First, the FDIPP design is not based on particular cryptographic algorithms. Instead, it allows the usage of any other cryptographic algorithms that provide similar functionality at the same security level with similar or less computational complexity. Second, the currently proposed design of FDIPP uses RSA only for key exchange, not for data transmission. This implies a low computational overhead as its usage is limited to transfer $SK_{i-AS}$ and $CK_{Cj}$, which does not frequently happen. Moreover, the additional delay introduced by using AES in encrypting/decrypting data packets does not hinder the ability of FDIPP to meet the latency requirements of IEC 61850-90-1. It has been shown in [49] that the usage of AES-128 bit in encryption and decryption by a resource-constrained WiFi router and a WiFi network card, respectively, increases the transmission time only by 12% compared with no encryption case, whereas increasing the key length of AES to 256 bits increases the latency of 128-bit AES by around 25% as presented in [50]. This represents an overall latency increase of 15%, which is insignificant given that the FDIPP end-to-end delay in the 4-gateway scenario is around 7 ms (3 ms below the IEC-61850-90-1 strictest delay constraint). The speed of AES encryption can also be significantly enhanced by offloading the encryption/decryption operations to a fast central processing unit (CPU) [51]. This notably enhances the implementation of network routers and dramatically reduces the encryption speed of AES to the order of microseconds [51]. Furthermore, the measurement results presented in [28] reveal that the computational latency for one-way hash function and message authentication code is around 0.1 ms. Since secondary substation routers, backbone routers, and gateways in the proposed architecture are not resource-constrained (in terms of processing or power), the computational latency does not significantly impact the network performance while FDIPP is in operation.

Apparently, using small SS clusters (clouds) leads to better performance during the key transfer phase. However, as the number of gateways increases, the Gateway-PS packet delay increases due to using more paths with more intermediate nodes, which increases the SSBC traffic volume. It is worth noting that the results are obtained using AODV, which is a standard non-QoS-aware routing protocol. Employing a QoS-aware routing protocol is anticipated to be able to achieve a similar packet delay and loss performance with a larger number of secondary substations.

#### 5.4.2. Execution Time for Node and Peer Authentication Phases

The execution time of the Node Authentication phase grows linearly with the number of nodes in the network since the authenticator shares the wireless channel with its one-hop neighbors (the supplicants). Therefore, it authenticates the neighbors one by one to avoid packet collisions.

The execution time of this phase can be approximately (ignoring the IEEE 802.11 average backoff time) estimated as

(3) $$T_{NS}\approx N\;E\left[N_{H}\right]\left(T_{S1}+T_{S2}+T_{S3}+T_{S6}+T_{S7}\right)$$

where *N* is the number of nodes (secondary substations or SSBC routers), $E[N_{H}]$ is average number of hops from a node to a PS, and $T_{Si}$ (i∈{1,2,3,6,7}) is the time to send the messages of Step 1, 2, 3, 6, and 7 as mentioned in Section 4.2, respectively.

It can be calculated as

(4) $$T_{Si}=T_{PHY}+\frac{H_{TCP}+H_{MAC}+L_{Si}}{R_{C}}+SIFS+T_{ACK}+DIFS,$$

where $H_{TCP}$ is the TCP protocol header size (20 Bytes) and C∈{SSBC,SSC}. The size of message *i* in Bytes ($L_{Si}$) varies, where $L_{S1}=L_{S2}=1$, $L_{S3}=L_{S7}=32$, $L_{S6}=48$, assuming 128 bits for keys and nonces, and 256 bits for hashing. Step 4, 5, 8, and 9 are not included as they are performed over a wired connection, whereas Step 10 belongs to the key transfer phase.

Using the parameters of Table 2, assuming the usage of four gateways, *N* is equal to 94 nodes (50 SSBC nodes and 44 SSC nodes and gateways), and $E[N_{H}]=3$ [52] for each of the SSBC and the SSC (from a gateway to an SS), which leads to $T_{NS}$ of around 45 ms including computational latency. This implies a fast convergence time of this phase, although it grows linearly with the number of nodes, as the network clouds are not loaded with any traffic during its execution.

The Peer Authentication phase is performed before a node can transmit data to any of its neighbors. Once the neighbor is authenticated, it shall be used later to forward the source node’s data over any route that involves this neighbor. The phase includes a short procedure of exchanging four short messages between two nodes, namely, two nonces (assumed 16 Bytes each) and the HMAC of these nonces (assumed 32 Bytes each), which leads to insignificant delay time given the high data rate of WiFi nodes in an SSC (54 Mb/s).

## 6. Conclusions

The paper presents a scalable and security-aware WiFi-based smart grid communication architecture that suits NIST guidelines for smart grid distribution systems. The proposed architecture specifically targets inter-substation communication in the low voltage network among secondary substations and between primary and secondary distribution substations. The proposed architecture groups secondary substations, within a small region, in a wireless mesh cloud that connects to the primary substation via a gateway and a wireless backbone cloud. The architecture scales by increasing the number of secondary substation clouds connected to the backbone.

Moreover, the paper introduces a detailed design of the FDIPP protocol that works over the proposed architecture to provide data confidentiality and integrity, protecting the system from wireless communication vulnerabilities. In addition to encrypting all exchanged packets, the FDIPP protocol authenticates every wireless node in the network, either it is a backbone router, a gateway, or a substation. Furthermore, the FDIPP management of security keys matches the network architecture design by exchanging two different types of keys, namely, cloud keys for routing messages and session keys for end-to-end data message exchange. This makes the design of FDIPP flexible to employ any commonly used wireless routing protocol in the secondary substation cloud or the backbone cloud, even if it is not security-aware such as AODV.

Comprehensive computer simulations show that the proposed network architecture can meet any stringent packet delay requirement of IEC 61850-90-1 for inter-substation communication with very low packet loss probability even during the FDIPP key exchange with the careful scaling of the number and size of secondary substation clouds.

## Figures and Tables

**Table 1:** IEC 61850-90-1 Delay Constraints [5].

Message Type	Delay Constraint (ms)	Usage
Type 1A	3–10	Fault isolation and protection (e.g., trip command)
Type 1B	2–100	Normal (routine communication) and other fast messages
Type 4	3–10	Raw data
Type 2	100	Monitoring and readings transfer (medium speed)
Type 3	500	Low speed data transfer
Type 6	1000	File transfer

**Table 2:** IEEE 802.11 system parameters.

System Parameter	Value
MAC Header $H_{MAC}$	208 bits
$T_{PHY}$	26 μs
$T_{ACK}$	5.583 μ
MAC Slot Time	20 μs
Short Inter-frame Space (SIFS)	10 μs
Distributed Inter-frame (DIFS)	50 μs
SSBC Number of Nodes	50
Data Rate SSC $R_{SSC}$	54 Mbps
Data Rate SSBC $R_{SSBC}$	300 Mbps
